# Structure of the Glycosyltransferase Ktr4p from *Saccharomyces cerevisiae*

**DOI:** 10.1371/journal.pone.0136239

**Published:** 2015-08-21

**Authors:** Dominik D. D. Possner, Magnus Claesson, Jodie E. Guy

**Affiliations:** 1 Department of Medical Biochemistry and Biophysics, Karolinska Institutet, Tomtebodavägen 6, S 171-77, Stockholm, Sweden; 2 Department of Biochemistry and Biophysics, Stockholm University, Svante Arrhenius väg 16C, S 106-91, Stockholm, Sweden; Monash University, AUSTRALIA

## Abstract

In the yeast *Saccharomyces cerevisiae*, members of the Kre2/Mnt1 protein family have been shown to be α-1,2-mannosyltransferases or α-1,2-mannosylphosphate transferases, utilising an Mn^2+^-coordinated GDP-mannose as the sugar donor and a variety of mannose derivatives as acceptors. Enzymes in this family are localised to the Golgi apparatus, and have been shown to be involved in both N- and O-linked glycosylation of newly-synthesised proteins, including cell wall glycoproteins. Our knowledge of the nine proteins in this family is however very incomplete at present. Only one family member, Kre2p/Mnt1p, has been studied by structural methods, and three (Ktr4p, Ktr5p, Ktr7p) are completely uncharacterised and remain classified only as putative glycosyltransferases. Here we use *in vitro* enzyme activity assays to provide experimental confirmation of the predicted glycosyltransferase activity of Ktr4p. Using GDP-mannose as the donor, we observe activity towards the acceptor methyl-α-mannoside, but little or no activity towards mannose or α-1,2-mannobiose. We also present the structure of the lumenal catalytic domain of *S*. *cerevisiae* Ktr4p, determined by X-ray crystallography to a resolution of 2.2 Å, and the complex of the enzyme with GDP to 1.9 Å resolution.

## Introduction

Ktr4p is a member of the Kre2/Mnt1 family of glycosyltransferases. Glycosyltransferase enzymes catalyse glycosidic bond formation by transferring a carbohydrate moiety from an activated ‘donor’ sugar substrate, most frequently a nucleoside diphosphate sugar, to an acceptor molecule which may be another sugar, a protein or nucleic acid, or a small molecule such as an antibiotic. They constitute a large and diverse class of enzymes, with more than 190,000 identified in sequenced genomes according to the CAZy database, (www.cazy.org)[1], and these are classified as belonging to more than 90 sequence-based families. The Kre2/Mnt1 family (classified as glycosyltransferase family 15 by the CAZy database) currently consists of nine members in yeast, and this family of enzymes play roles in the glycosylation of newly-synthesised proteins. There are no homologues of the proteins in higher eukaryotes.

In the yeast *Saccharomyces cerevisiae*, as in other eukaryotes, N-glycosylation of proteins begins in the ER, where a core glycan is assembled on a lipid carrier and subsequently transferred onto the asparagine residue of the target protein by an oligosaccharyltransferase complex. This core is then modified in the Golgi apparatus, where the diversity of N-glycan structures is achieved by a series of glycosidase and glycosyltransferase enzymes (reviewed by [2,3]). In yeast there are two main types of N-glycan structures: large mannan structures, consisting of a backbone of about 50 mannoses and short side branches, are frequently found on structural cell-wall proteins while glycoproteins destined for the internal compartments of the cell have much smaller mannose-based structures. O-linked glycosylation is also present in *S*. *cerevisiae*, and generally consists of a linear chain of between 1 and 5 mannose residues. In this case the first mannose is added in the ER and the remainder are attached in the Golgi apparatus by mannosyl transferase enzymes. Members of the Kre2/Mnt1 family have been shown to play roles in both N- and O-linked glycosylation in *S*. *cerevisiae*, and several have been specifically linked to the synthesis of cell wall glycoproteins, with mutations in the genes encoding these enzymes resulting in disturbance or weakening of the cell wall (reviewed in [4]).

So far, six enzymes of the Kre2/Mnt1 family have been enzymatically characterised, with Kre2p/Mnt1p being the best studied. Yeast Kre2p/Mnt1p catalyses the addition of the second and third α-1,2-linked mannose residues in linear O-linked oligosaccharides, and has also been shown to be involved in the synthesis of the outer chains of N-linked oligosaccharides [5–7]. Kre2p/Mnt1p is the only member of the family for which a structure has been determined. The crystal structure of the catalytic domain [8] revealed Kre2p/Mnt1p to consist of one Rossmann-fold type domain tightly packed against a second domain of α/β-structure, and thus to belong to the GT-A fold family of glycosyltransferases, rather than the GT-B fold family which has two, less tightly-packed, Rossmann-like domains with the active site in a cleft between them.

Of the other members of the Kre2/Mnt1 family, Ktr1p, Ktr2p, Ktr3p and Yur1p have been shown to have similar and partially redundant mannosyltransferase functions [4], while Ktr6p is a mannosylphosphate transferase [9,10]. Although studies of this family of enzymes have primarily been performed on the *S*. *cerevisiae* proteins, a closely homologous enzyme from the family is found in the human pathogen *Candida albicans*. This protein, known as CaMnt1p, has been implicated in both the adhesion and virulence properties of *C*. *albicans* [11], and structural and functional studies of the Kre2/Mnt1 family of proteins could therefore be important for the development of antifungal agents.

Very few studies have been performed on Ktr4p itself, and its exact role is not yet known although the fact that it exhibits a 32% sequence identity to Kre2p/Mnt1p, with several of the residues responsible for binding the nucleotide and Mn^2+^ in Kre2p/Mnt1p conserved, implies that it is likely to have a similar function to the α-1,2-mannosyltransferase. The homologous Ktr4 protein in the filamentous fungi *Beauvaria bassiana* has been studied, and its deletion has been shown to lead to growth defects, a decrease in cell wall components, reduced tolerance to stress and lower virulence [12], reinforcing the potential importance of the Ktr4p protein.

Ktr4p, as the other members of the Kre2/Mnt1 family, is a type II membrane protein with a short N-terminal cytosolic tail and a large lumenal catalytic domain of 40 kDa, which is separated from the membrane by a stalk domain of 11 kDa. It is found primarily in the Golgi apparatus, and this localisation depends on interaction between Ktr4p and the complex of ER-vesicle proteins Erv46p and Erv41p [13–15].

In order to increase our understanding of Ktr4p and its functions in the cell, and to provide a greater understanding of the structures of the Kre2/Mnt1 family glycosyltransferases, we have solved the structure of the lumenal domain of Ktr4p from *Saccharomyces cerevisiae*. Here we present structures of the apo-enzyme and the GDP-complex. We show that the enzyme belongs to the GT-A fold family, although the 2-domain structure that is a main feature of this fold is partly concealed in Ktr4p by the positions of two helices from the N-terminal stalk domain. Using *in vitro* enzyme activity assays we also provide experimental support for the predicted glycosyltransferase activity of Ktr4p, and show it to have a preference for methyl-α-mannoside out of the tested acceptor substrates.

## Materials and Methods

### Cloning and Expression Screening

Based on secondary structure predictions and multiple sequence alignments with other members of the Kre2/Mnt1 family (GT family 15), twelve different constructs of Ktr4p were designed. These constructs spanned the lumenal domain of the protein from Asn-33 to Tyr-464; all included the predicted catalytic domain, with some also incorporating the membrane-proximal stalk domain. Each construct was amplified by PCR from genomic *S*. *cerevisiae* S288c DNA. The PCR products were then cloned into the pNIC28-Bsa4 vector by applying the ligase independent cloning method [16], thereby introducing a His_6_-purification tag followed by a tobacco etch virus (TEV) protease cleavage site N-terminal of each Ktr4p construct. The presence and integrity of each construct was confirmed by sequencing.

For expression tests, *E*. *coli* Rosetta-gami 2 (DE3) cells were transformed with the plasmids containing the different constructs. 1ml TB supplemented with 10% (v/v) glycerol was then inoculated with a single colony and grown at 37°C until the OD_600_ reached 0.4. At this point the temperature was reduced to 18°C, before expression was induced by addition of IPTG to a final concentration of 0.5 mM and allowed to continue overnight. Cell cultures were pelleted, resuspended in lysis buffer 1 (100 mM HEPES; pH 8.0 with 500 mM NaCl, 10 mM MgSO_4_, 10% (v/v) glycerol, 0.1% (w/v) DDM, 10 mM imidazole, 1 mgml^-1^ lysozyme, 0.125 U Benzonase, 0.5 mM TCEP, cOmplete protease inhibitor cocktail (Roche Diagnostics)) and lysed by freezing at -80°C and thawing. The lysates were subsequently pelleted and the soluble protein expression resulting from each construct was detected by SDS-PAGE analysis.

The construct that was ultimately chosen to produce protein for structure determination was the longest that produced soluble protein. This corresponded to residues Asn-33—Tyr-464 of the protein, and was amplified using the primers 5'-TACTTCCAATCCATGAATGAGAACTATTTGCAAGCAG-3' and 5'-TATCCACCTTTACTGTCAATACATTTCTAACTCTTCCTCAG-3'.

### Large Scale Protein Production

For large scale protein expression, 6 l of TB media supplemented with 10% (v/v) glycerol was inoculated with a pre-culture (1:500 dilution) and incubated at 37°C until an OD_600_ of 0.6 was reached. The temperature was then reduced to 21°C; induction was performed by addition of IPTG to a final concentration of 0.5 mM, and expression was allowed to continue in these conditions overnight before the cells were harvested by centrifugation.

Cell lysis was performed by sonication in lysis buffer 2 (10 mM HEPES; pH 7.4 with 300 mM NaCl 10 mM imidazole, 5% (v/v) glycerol, 0.04 mgml^-1^ DNAse, 0.004 mgml^-1^ lysozyme and cOmplete protease inhibitor cocktail (Roche Diagnostics)). The lysate was cleared by centrifugation (30 min at 18000 g, 4°C) and the His_6_-tagged Ktr4p protein was then purified by IMAC using 1 ml Ni-NTA agarose (Qiagen). The column was washed twice with 3 column volumes (CV) of washing buffer (10 mM HEPES; pH 7.4 with 300 mM NaCl, 20 mM imidazole); bound protein was eluted with 5 CV of elution buffer (10 mM HEPES; pH 7.4 with 300 mM NaCl, 500 mM imidazole) and the eluted protein was subsequently treated with TEV protease and simultaneous dialysis against TEV protease cleavage buffer (10 mM HEPES; pH 7.4 with 150 mM NaCl, 0.5 mM TCEP, overnight at 4°C) to remove the His_6_-purification tag. The solution was passed once more through the IMAC resin for removal of the His_6_-tagged TEV protease and the flow-through was collected. Size exclusion chromatography was performed in 10 mM HEPES; pH 7.4 with 150 mM NaCl using a Superdex 200 16/60 column (GE Healthcare). The protein eluted from the column at volumes corresponding to both monomer and dimer with a ratio of ~2:1; the fractions contained within the major peak corresponding to the monomeric protein were pooled, and the protein solution was subsequently concentrated to 15 mgml^-1^ using a Vivaspin 6 concentrator (10 kDa cut-off, Sartorius) for crystallisation. Homogeneity and monodispersity of the final sample was assessed using SDS-PAGE and dynamic light scattering (DLS), respectively, and circular dichroism experiments produced a mainly α-helical spectrum, indicating that the protein was folded (results not shown).

### Crystallisation and Structure Determination

Initial crystallisation screening was performed with commercially available crystallisation screens using the vapour diffusion method in Corning #3350 plates. Sitting drops of 300 nl final volume were set up using a Mosquito dispenser and equilibrated against 50 μl of mother liquor at 4°C.

Initial crystallisation conditions were then reproduced and optimised in larger drops. The optimised condition that yielded crystals for data collection was 0.1 M sodium cacodylate buffer, pH 6.5; 0.2 M Ca(OAc)_2_; 18% (w/v) PEG 8000. Crystals were produced in this condition using 1.5 μl sitting-drops in the MRC crystallisation plate (Molecular Dimensions), by mixing 1 μl protein solution with 0.5 μl of mother liquor and equilibration of this drop against 75 μl of mother liquor at 4°C. Streak seeding was necessary to obtain crystals in the larger volume; immediately after the drops were set up, previously-obtained crystals were touched with a cat whisker which was then drawn through the drops. The first crystals were observed after approximately 3–5 days incubation.

To obtain crystals of the ternary complex, Ktr4p at a concentration of 15 mgml^-1^ was pre-incubated for 1 h with 5 mM GDP and 5 mM MnCl_2_, followed by the same crystallisation procedure.

The crystals were cryoprotected by adding glycerol to the crystallisation drop to a final concentration of 15% (v/v), prior to freezing by plunging them into liquid nitrogen. X-ray diffraction data were collected at 100K from the frozen crystals using the European Synchrotron Radiation Facility (ESRF) beamlines ID23-1 and ID23-2. Data corresponding to the apo structure were collected at the microfocus beamline ID23-2, which is equipped with a PILATUS 2M detector; 300° of data were collected at a wavelength of 0.8726 Å and an oscillation angle of 0.15°. Data corresponding to the complex structure were collected on beamline ID23-1, which is equipped with a PILATUS 6M detector; 360° of data were collected at a wavelength of 0.9724 Å and an oscillation angle of 0.15°. All data were processed using XDS [17] and AIMLESS [18] from the CCP4 Program Suite [19], and the datasets were truncated at 2.2 Å and 1.9 Å resolution, respectively, based on R_merge_ statistics.

Molecular replacement was performed using Phaser [20]. The Kre2p/Mnt1p structure (PDB 1s4n) [8], was used as the search model for the apo structure, whereas the apo structure was used as the search model for the structure of the ternary complex. Model-building was performed in Coot [21] and iterative local NCS refinement using REFMAC5 [22]. For the two datasets, 5% of independent, randomly selected reflections were used to monitor the R_free_. Towards the end of refinement, water molecules were added automatically in Coot before being checked manually and refined. In the course of structure refinement, it became apparent that residual density at several sites could not be accounted for by the protein or solvent. Density corresponding to three metal ions was observed on the surface of monomer B in the apo dataset. These metal ions were modelled as calcium in the deposited structure, because calcium is present in the crystallisation condition and it gave a good fit to the density as well as B-factors that are consistent with those of neighbouring residues. Further density on the surface of each monomer in the GDP complex was assigned as acetic acid, based on the shape of the electron density and the hydrogen bonding pattern. These ligands, as well as the GDP in the active site of the complex data, were imported within Coot and their positions were refined with REFMAC5.

The atomic coordinates and crystallographic data have been deposited with the Protein Data Bank, with accession codes **5a08** for the apo structure and **5a07** for the GDP-complex structure.

Ternary structures and crystal packing interfaces were analysed using the PISA server at the European Bioinformatics Institute [23]. Analysis of the structure was assisted by output from the PDBsum Generate webserver [24,25] and database searches for structural homologues to Ktr4p were performed with DALI [26]. Figures were prepared in PyMOL [27].

### Biochemical Assays

The transfer activity of Ktr4p was determined *in vitro* using the Glycosyltransferase Activity Kit from R&D Systems, following the instructions supplied by the manufacturer. In this coupled assay the leaving GDP of the mannosyltransferase reaction is further hydrolysed by the calcium-dependent nucleotidase ENTPD3/CD39L3 giving rise to free inorganic phosphate, which can be detected by the malachite green reagent. The reaction was initiated by the addition of 50 μM GDP-Mannose to 10 μM Ktr4p and 500 μM acceptor substrate (methyl-α-mannoside, α-1,2-mannobiose or mannose) in 25 mM Tris-HCl; pH 7.5, with 10 mM CaCl_2_, 10 mM MnCl_2_ at 20°C and stopped at different time points by the addition of the malachite green development solution. All reactions were performed in triplicate.

## Results

### Protein production and structure determination

Of all designed protein constructs, it was decided to use the longest soluble one, covering the complete lumenal domain of Ktr4p (residues Asn-33—Tyr-464), for crystallisation trials and enzymatic assays; this construct is referred to simply as ‘Ktr4p’ from this point onwards.

After size exclusion chromatography, the final purification step, the Ktr4p protein was judged by SDS-PAGE and DLS to be both pure and monodisperse (results not shown). Initial crystallisation trials with the recombinant Ktr4p protein gave small but promising crystals in one crystallisation condition, which was optimised to produce the crystals used for data collection. In the optimised condition, single crystals with bi-pyramidal morphology grew after 3–5 days incubation in drops with a 2:1 ratio of protein:mother liquor.

X-ray diffraction data for the apo structure was collected to 2.2 Å resolution and indexed in the space group P2_1_2_1_2_1_ with the unit cell dimensions a = 60.2 Å, b = 102.4 Å, c = 156.9 Å, α = β = γ = 90°. The Matthews' coefficient [28] suggested the presence of two molecules per asymmetric unit (corresponding to a solvent content of 51%). Molecular replacement in Phaser, using the Kre2p/Mnt1p (PDB id 1s4n) monomer structure as search model, successfully placed two monomers, and after rebuilding the sequence the structure was refined to R/R_free_ values of 0.163/0.202. The GDP complex data was indexed in space group P2_1_2_1_2_1_ to 1.9 Å resolution, with unit cell dimensions a = 61.2 Å, b = 102.6 Å, c = 162.7 Å, α = β = γ = 90°. The complex structure was determined using the refined apo structure as search model, and was refined to R/R_free_ values of 0.156/0.191. The data collection and refinement statistics for each dataset are summarised in Table 1.

**Table 1:** Data collection and refinement statistics.

	Ktr4p apo structure	Ktr4p-GDP complex
**Data collection**		
Beamline	ESRF ID23-2	ESRF ID23-1
Wavelength (Å)	0.8726	0.97241
Space group	P2_1_2_1_2_1_	P2_1_2_1_2_1_
Cell axes (Å)	60.19, 102.37, 156.91	61.215, 102.62, 162.65
Cell angles (°)	90, 90, 90	90, 90, 90
Resolution (Å)	50–2.2 (2.28–2.20)	50–1.9 (1.94–1.90)
R_merge_	0.127 (0.651)	0.104 (0.867)
Mean I/σI	13.8 (3.0)	15.5 (2.3)
Mean CC_1/2_	0.997 (0.856)	0.999 (0.833)
Completeness (%)	99.7 (97.0)	99.1 (98.9)
Multiplicity	7.8 (8.0)	9.8 (9.2)
Number of reflections	387144 (34874)	790203 (41691)
Number of unique reflections	49596 (4381)	80667 (4508)
Wilson B-factor (Å^2^)	10.5	17.5
**Refinement**		
Resolution (Å)	50–2.2	50–1.9
R/R_free_	0.163/0.202	0.156/0.191
Number of non-hydrogen atoms (protein)	7081	7278
Mean B value (Å^2^)	25.0	26.5
Number of waters	498	577
rmsd bond lengths (Å)	0.018	0.020
rmsd bond angles (°)	1.678	1.832
**Ramachandran plot**		
Residues in favoured regions (%)	97.8	98.2
Residues in allowed regions (%)	2.2	1.8
Residues in disallowed regions (%)	0	0

Values in parentheses are statistics for the highest resolution shell.

$$R=\frac{\sum_{hkl}\left|F_{obs}\left(hkl\right)-F_{calc}\left(hkl\right)\right|}{\sum_{hkl}F_{obs}\left(hkl\right)}$$
$$R_{merge}=\frac{\sum_{hkl}\sum_{i=1}^{n}\left|I_{i}\left(hkl\right)-\bar{I}\left(hkl\right)\right|}{\sum_{hkl}\sum_{i=1}^{n}I_{i}\left(hkl\right)}$$

Both the native and complex structures are well-defined in the electron density map, and the density for the protein is continuous with the exception of residues 224 to 230 in chain B of the apo form, which corresponds to a short disordered loop. These residues are visible in chain A and the slightly higher resolution complex structure. Also missing are residues of the extreme N-terminus (residues 33–69 in chain A of the complex and residues 33–70 in chain B of the complex as well as both molecules of the apo structure), which belong to the stalk separating the protein from the membrane. No significant differences are visible between the two chains in the asymmetric unit in either the apo-form or complex structure (maximum RMSD < 0.45 Å over all Cα atoms).

### Structure of the Ktr4p monomer

**Fig 1:**
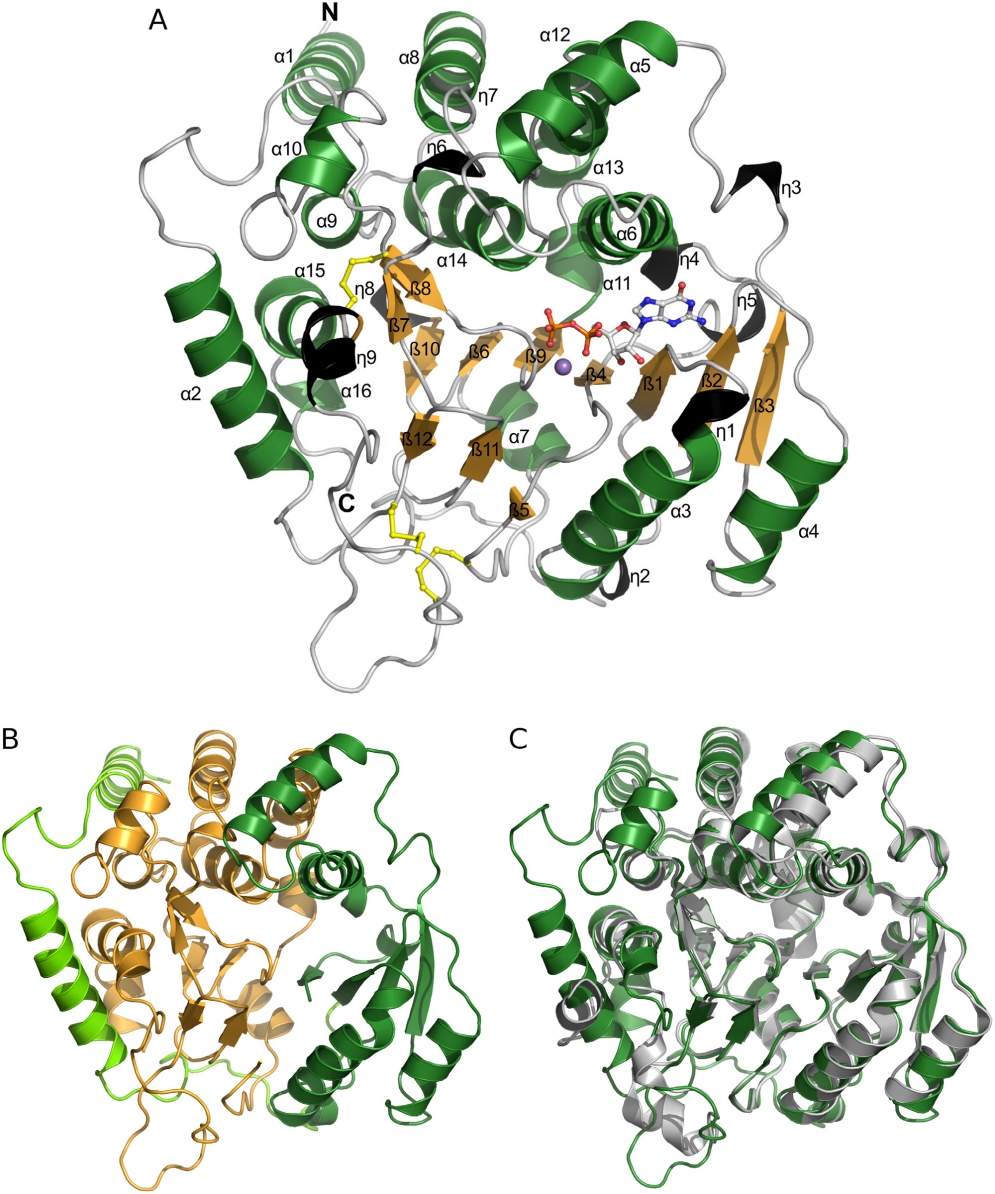
The structure of Ktr4p. Panel A shows the structure of Ktr4p in complex with GDP and Mn^2+^. α-helices are coloured in green, 3_10_-helices in black and β-strands in orange, and all secondary structure elements are numbered. The GDP is shown in ball-and-stick representation, as are the cysteines forming disulfide-bonds. Panel B shows a cartoon representation of Ktr4p coloured by its subdomains. The two N-terminal helices (in light green) are bridging over the C-terminal subdomain (orange) and connecting to the N-terminal subdomain (dark green). Panel C shows the Ktr4p structure (green) superimposed on that of the homologous Kre2p/Mnt1p (grey).

The structure of the Ktr4p monomer is comprised of a mixed α/β-fold, containing 12 β-strands, 16 α-helices and 9 short stretches of 3_10_-helix (secondary structure assignments determined by PDBsum) [24,25]. At the core of the structure is a seven stranded β-sheet of mixed type, comprised of β-strands β3, β2, β1, β4, β9, β6, β10, in which all strands are parallel with the exception of β9. This β-sheet is surrounded by α-helices, and is flanked by two small β-sheets, one consisting of two short antiparallel strands (β7, β8) and one of 3 short antiparallel strands (β5, β11, β12). The structure is shown in Fig 1A.

Nucleotide-sugar dependent glycosyltransferases belong to two fold types; GT-A, containing two β-α-β domains of differing size which are so closely packed that a continuous central β-sheet is formed, and GT-B, which consist of two Rossmann-like domains that are less closely packed and contain the active site in a cleft between them (reviewed by [29]). Ktr4p, with its large central β-sheet, belongs to the GT-A fold class of glycosyltransferases. However, the construct used for crystallisation encompasses the membrane-proximal stalk domain in addition to the catalytic domain. By homology, the N-terminal subdomain would encompass β-strands 1–4 and their flanking helices (from residue 135 at the N-terminus of β-strand 1 to residue 261 at the C-terminal end of β-strand 4), while the C-terminal subdomain would begin from residue 266 at the start of β-strand 5, incorporating β-strands 5–12 and their flanking helices. In our structure, although the extreme N-terminus is not visible, part of the stalk domain, corresponding to helices α1 and α2, is clearly defined in the electron density. These helices protrude from the N-terminal subdomain of the protein and fold over the surface of the C-terminal subdomain, which has the effect of making the protein appear more like a large single domain structure (Fig 1B). Helices α1 and α2 are relatively tightly packed against the main body of the protein, with 4 salt bridges present in addition to hydrogen bonds and hydrophobic interactions, but whether these two helices would sit in this position in the native, membrane-bound protein is impossible to determine from the currently-available data. They are involved in crystal packing and it is possible that their position could be influenced by this fact, and that the stalk domain might protrude further from the body of the molecule to separate it from the membrane in the intact protein.

Three disulfide bonds are present in the C-terminal subdomain of the protein, between residues Cys-269 – Cys-427, Cys-345 – Cys-447 and Cys-417 – Cys-431; these likely provide significant additional stability to the subdomain. The active site is located in a long cleft, with one side of the cleft formed from an edge of the central β-sheet (primarily the C-terminal ends of β-strands 2, 1, and 4) as well as the hairpin between β11 and β12, and the other side formed largely by residues from α-helices α8 and α16. The apo structure of Ktr4p contains no metals in the active site, but density corresponding to three metal ions was observed on the surface of monomer B. These metal ions, modelled as calcium in the deposited structure are grouped close together and are coordinated by the sidechains of Asp-77 and Asp-81, Asp-308 and Glu-311, and His-84 and Glu-311, respectively. They are involved in the formation of crystal contacts and are not near the active site; it is therefore likely that they are merely a result of crystal packing.

### Comparison to the Kre2p/Mnt1p structure

A search for structural homologues of Ktr4p was performed using the Dali server [26] and, as would be expected from sequence identity of 32%, the closest structure identified in the PDB was that of Kre2p/Mnt1p (e.g. PDB id 1s4p), with RMSD of 1.7Å over 303 residues. Significantly lower structural similarity was reported between Ktr4p and other structures in the PDB. Galactosyltransferase LgtC from *Neisseria meningitides* (e.g. PDB id 1ss9), with RMSD 3.8Å over 280 residues, and human Fucosylgalactoside-α-N-acetylgalactosaminyltransferase (e.g. PDB id 3v0o), with RMSD 3.5Å over 290 residues, were the next-highest matches.

The catalytic domain structure of Ktr4p is very similar to that of Kre2p/Mnt1p; if only 235 Cα atoms of the catalytic domain are used for superimposition, then the monomers can be superimposed with an RMSD of 1.2 Å (using LSQ superimposition as implemented in Coot [21]). The superimposed monomers are shown in Fig 1C. The biggest difference observed is between the N-termini of the two proteins. As mentioned previously, the Ktr4p construct that has been used for crystallisation included both the stalk domain and catalytic domain, and part of the stalk domain is visible in the structure as two long helices at the N-terminus preceding β-strand β1. In the Kre2p/Mnt1p structure (PDB id 1s4n) there are only five residues visible at the N-terminus before β1. The Kre2p/Mnt1p structure does include a short disconnected helix which the authors assign as part of the stalk region (residues 103–114), but the position of this does not coincide with α1 or α2 in Ktr4p. In fact our helix α2 partly overlaps the positions taken in the Kre2p/Mnt1p structure by a short 3_10_ helix at the extreme C-terminus (η9), and residues 313–318. Helix η9 has no equivalent in Ktr4p, as the C-terminus is slightly shorter, and residues 313–318 are part of a loop which has a different conformation in Ktr4p. Superimposition of the Kre2p/Mnt1p and Ktr4p monomers also reveals differences in the structures of several further loops, while the remainder of the secondary structure is largely conserved between the two proteins. The central large β-sheet, in particular, is extremely well conserved. The authors of the Kre2p/Mnt1p structure note that the three disulfide bonds in the C-terminal subdomain of Kre2p/Mnt1p appear to be conserved across the family in *S*. *cerevisiae* with the exception of Ktr4p, in which Cys-406 of Kre2p/Mnt1p is replaced with a glycine residue. Our structure reveals that while this is the case, with Gly-429 being the glycine in question, the equivalent disulfide bond is instead formed with the closely-positioned Cys-427. There are therefore still three disulfide bonds in Ktr4p, as expected in all other members of the family, and no significant difference results to the overall structure.

Another difference between the Ktr4p and Kre2p/Mnt1p structures is the way in which the monomers associate. In the Kre2p/Mnt1p structure the monomers associate into relatively tight dimers, which bury an exposed surface area of 1255 Å^2^ of each monomer and are classified by the PISA server as likely to be physiologically relevant. The Ktr4p monomers also associate into symmetrical dimers within the crystal structure, with part of the interface the same as that seen in Kre2p/Mnt1p. The monomer-monomer interface in Ktr4p is however significantly less extensive, with only 761 Å^2^ of solvent-accessible surface buried. This interface in Ktr4p is mainly built by interactions between helices α3 and α4 of one chain with the loops between β11-β12 and β12-η9 of the opposing chain, while the interface in in Kre2p/Mnt1p extends down the length of the protein. In both dimeric structures the GDP-binding pocket is surface exposed, but in Kre2p/Mnt1p the pocket is accessible *via* a narrow channel from the solvent, whereas Ktr4p would provide a more open environment for the ligand to enter the active site. The relevance of a dimeric state of the protein remains, however, unclear. Our SEC studies indicate that the Ktr4p protein exists as both monomers and dimers in solution, but the monomer peak is the largest and it was therefore the fractions from this that were used for crystallisation. Furthermore, we are working with an isolated domain of a membrane protein, which makes it difficult to predict the oligomeric state of the native form.

### The Ktr4p-GDP complex structure and active site

**Fig 2:**
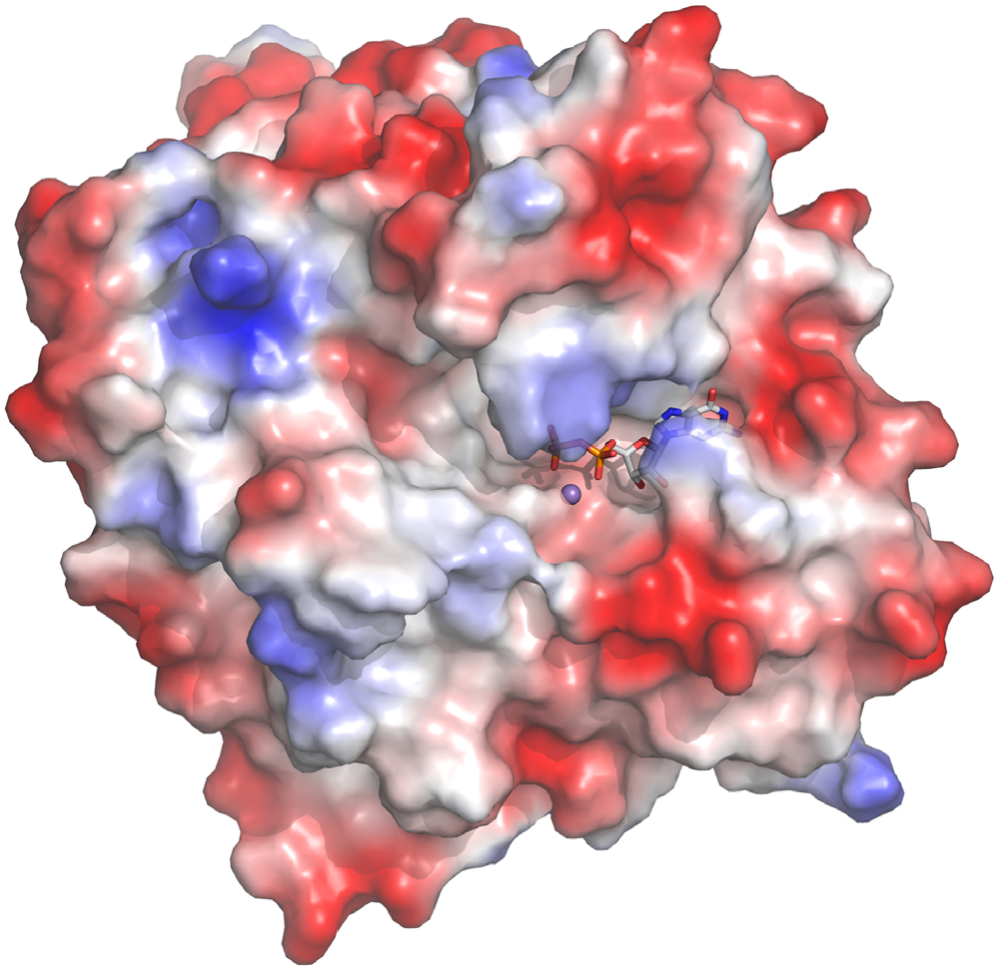
A surface representation of Ktr4p, showing the shape and position of the substrate-binding cavity. The surface is coloured by electrostatic potential, as calculated using PyMol, and the GDP and Mn^2+^ of the complex structure are added in ball-and-stick representation to indicate the location of the active site.

We have formed the complex of Ktr4p and GDP and Mn^2+^ by soaking crystals of the apo-form, and solved the structure of this complex to 1.9 Å resolution. Both ligands bind in the previously described active-site cleft, which is situated on the surface of the protein, as seen in Fig 2. The complex structure shows that only slight changes occur in the Ktr4p structure upon ligand binding; the monomer structures of Ktr4p in the presence and absence of GDP and Mn^2+^ can be superimposed with a maximum RMSD of 0.37 Å over 386 Cα atoms (chain B of the apo structure superimposed on chain A of the complex structure). The most noticeable difference, apart from the GDP itself, is the presence of a Mn^2+^ ion bound in the active site of each monomer in the complex. The Mn^2+^ ion in the active site is hexahedrally coordinated. The metal ion interacts with the OE1 of Glu-262 at a distance of 2.2 Å, the NE2 of His-411 (2.4 Å), water molecules 1 and 2 (both at 2.4 Å distance), as well as the O1 of the α- (2.3 Å) and the O1 of the β-phosphate (2.2 Å).

**Fig 3:**
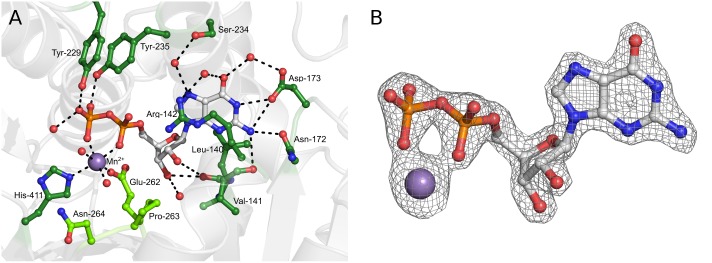
GDP binding in the complex structure. Panel A shows a ball-and-stick representation of GDP (white) and Mn^2+^ (purple) located in the active site and residues involved in their binding (green). Residues of the EPN-motif (Glu-262, Pro-263, Asn-264) are shown in light green. Panel B shows an Fo-Fc omit map corresponding to the ligand and the metal, contoured at 3σ, with GDP and Mn^2+^ depicted as ball-and-stick models.

The position of the GDP ligand is very well described by the electron density (Fig 3B), and its interactions with the protein can therefore be described with confidence. In addition to their interaction with the metal, the phosphate groups of the GDP make several interactions with the protein itself. O1 of the β-phosphate interacts with the OH of Tyr-229 at a distance of 2.7 Å, and O2 of the β-phosphate interacts with the OH of Tyr-235 at a distance of 2.7 Å. One water molecule is bound by both O3 of the β-phosphate and O2 of the α-phosphate, while O3 of the β-phosphate also binds two further water molecules. The ribose moiety of the GDP forms two hydrogen bonds with the protein; its O2’ interacts with the main chain oxygen of Leu-140 at a distance of 3.1 Å, and its O3’ interacts with the same atom at a distance of 2.7 Å as well as to one water molecule. The ribose moiety also makes van der Waals interactions with the protein, including Leu-140, Met-253 and Pro-263. The guanine moiety of the GDP makes hydrogen bonds to three residues of the enzyme *via* its nitrogen atoms; N1 interacts with OD1 of Asp-173 at 2.7 Å distance, while N2 interacts with the same atom (3.2 Å distance), OD1 of Asn-172 (2.9 Å) and the main chain O of Val-141 (3.0 Å). N7 of the guanine binds to a water molecule, which in turn interacts with the OH of Ser-234, and O6 binds to two waters which in turn bind to Asp-173 and Arg-142, respectively. There are also a number of van der Waals and stacking interactions with the guanine moiety, including residues Arg-142, Trp-204 and Met-238.

Only slight changes in sidechain conformation can be observed upon ligand binding in the active site, most notably the position of Arg-142 changes upon ligand binding to free space for the nucleotide base and interact with it *via* a stacking interaction, meaning the gross structure of the active site pocket is preformed in the proteins apo-state despite the absence of the Mn^2+^ ion. This is relatively unusual in glycosyltransferase enzymes; more commonly conformational changes (rearrangement of loops) are observed on binding of the donor, which are thought to help sequester the active site from solvent and potentially assist in product release [30]. Although it is possible that restricted flexibility of the protein in the crystalline state could influence the results of these soaking experiments, the lack of conformational rearrangement in Ktr4p on GDP-binding is consistent with what was previously observed in the Kre2p/Mnt1p structures in the presence and absence of GDP.

Ktr4p does not contain the DXD motif (where X represents any amino acid), which is found in many glycosyltransferases with the two acidic residues coordinating the essential divalent cation. This motif was previously thought to be completely conserved in glycosyltransferase enzymes that display the GT-A fold, and to be a ‘signature’ of the class, but more recently there have been several examples of GT-A proteins with variations in one or more of the residues in the motif (reviewed in [29]). It is not completely conserved in any of the Kre2 family, with five of the enzymes instead having the sequence EPD and the remainder displaying different variations. In Ktr4p the equivalent sequence is EPN (Glu-262, Pro-263, Asn-264), as seen in Fig 3A. In the complex structure Glu-262 is coordinating the active site Mn^2+^ ion *via* its OE1 atom, while Asn-264 is on the surface of the monomer and is indirectly interacting with the second chain of the ‘dimer’ *via* two water molecules. This interaction occurs in both chains of the symmetrical ‘dimer’.

### *In vitro* Ktr4p activity assays

**Fig 4:**
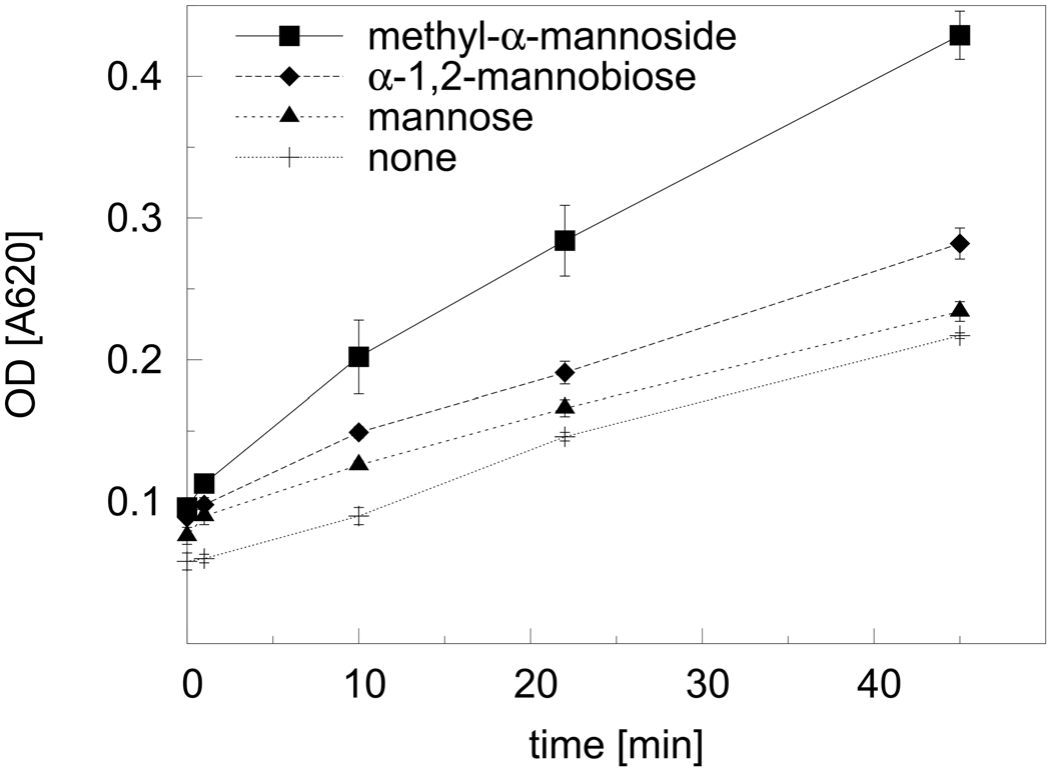
Activity of Ktr4p. The enzyme is active using methyl-α-mannoside (■) as acceptor substrate, and the signal observed using α-1,2-mannobiose (♦) and D-mannose (▲), respectively, is comparable to the background reading in the absence of acceptor substrate (+). The blank reading, measured in the absence of enzyme, has been deducted from all experimental readings.

Although sequence identity strongly suggested Ktr4p to be a glycosyltransferase enzyme, this catalytic activity had not yet been experimentally tested. We therefore performed *in vitro* activity assays with our purified recombinant protein to both confirm the putative function and to perform a preliminary investigation of substrate specificity. We used a coupled spectrophotometric assay, in which the hydrolysis of GDP-mannose by the glycosyltransferase is followed by enzymatic removal of the β-phosphate from the resulting GDP-moiety and subsequent quantification of inorganic phosphate. We used GDP-mannose as the sugar donor, and tested mannose, α-1,2-mannobiose and methyl-α-mannoside as potential acceptors; in the case of both donor and acceptor this was based on suggestions from sequence identity. Our results, shown in Fig 4, confirm that Ktr4p is an active mannosyltransferase enzyme. The fact that GDP-mannose is a suitable sugar donor is in agreement with our complex crystal structure which confirms that GDP, at least, can effectively bind to the protein. When comparing activity towards mannose, α-1,2-mannobiose and methyl-α-mannoside as acceptors, we observe the most efficient activity with methyl-α-mannoside (Fig 4). Reaction rates with α-1,2-mannobiose and mannose are lower, although both still appear to be slightly higher than seen in the absence of an acceptor. The fact that the enzyme can cleave the donor in the absence of an acceptor sugar is not surprising in light of the fact that our attempts to crystallise a GDP-mannose complex resulted in very good GDP density (Fig 3B) and little or no mannose density.

## Discussion

Despite extensive efforts to produce a structure of Ktr4p in complex with GDP-mannose, both by co-crystallisation and by soaking native crystals with GDP-mannose, we have not been able to observe the non-hydrolysed state of the ligand, but only the GDP moiety. Even soaking crystals in freshly-prepared GDP-mannose solution for the shortest possible amount of time did not make it possible to observe the intact ligand in the electron density, while the GDP moiety is always clearly visible and well-defined. A similar situation was reported by the authors of the Kre2p/Mnt1p structure, who were also unable to observe the non-hydrolysed ligand in their crystal structures [8]. With Ktr4p we have on several occasions observed weak electron density at a position juxtaposed to the β-phosphate of the nucleotide, which is at the base of a pocket that reaches from the active site to the solvent-accessible surface of the protein. This density (shown in S1 Fig) was not of sufficient quality to enable us to unambiguously model mannose although it is of approximately the correct size, and for reasons described below we believe it corresponds to the position of the acceptor-, rather than donor-sugar. As glycerol was used as cryoprotectant it is possible that this density could at least in part be attributed to glycerol substituting for the acceptor sugar, which has been observed in other glycosyltransferase structures. We therefore collected data from crystals in which the glycerol was substituted with mannose, PEG400 and Paratone N, respectively, but although density was still present in this position it was not improved by the excess of mannose and/or absence of glycerol.

Protruding from the same pocket in which the ‘acceptor’ density is observed, and adjacent to the β-phosphate and the manganese ion, is a cavity which is partially occupied by water molecules in our structure (waters 65, 75, 141 and 512 in the GDP complex). This pocket is of sufficient size and well-positioned to accommodate the carbohydrate moiety of the donor substrate, and it is in this part of the active site pocket that the carbohydrate moiety of the donor substrate has been observed in homologous GT structures [31–33]; it has also been suggested to accommodate the carbohydrate moiety of the donor substrate in Kre2p/Mnt1p [8]. Binding of the donor carbohydrate in this pocket would expose the anomeric carbon in a strained conformation suitable for transfer onto the acceptor substrate if the latter was located in the position of the observed electron density. Several suitable hydrogen bonding donors are present in the proposed donor sugar site, and we were therefore able to manually model both the donor GDP-mannose and an acceptor sugar using information gained from our GDP/Mn^2+^ complex structure together with information from binary and tertiary complexes of other GT-A proteins [8, 31–33] (S1 Fig). Detailed analysis of sugar binding, and mechanistic studies, will however require high-resolution crystal structures of the complexes with both donor and acceptor sugars.

Whether the lack of density for the mannose moiety of GDP-mannose in our structures is due to hydrolysis of the glycosidic bond of the donor substrate *in crystallo*, or an inherent flexibility of the mannose moiety in the bound ligand, is not possible to say for sure. However, our activity assays do confirm that Ktr4p in solution is capable of cleaving of the GDP-mannose even in absence of an acceptor. We do not observe significant cleavage in the absence of Ktr4p, confirming that it is an enzyme-mediated catalysis, which implies that the latter scenario is more likely.

**Fig 5:**
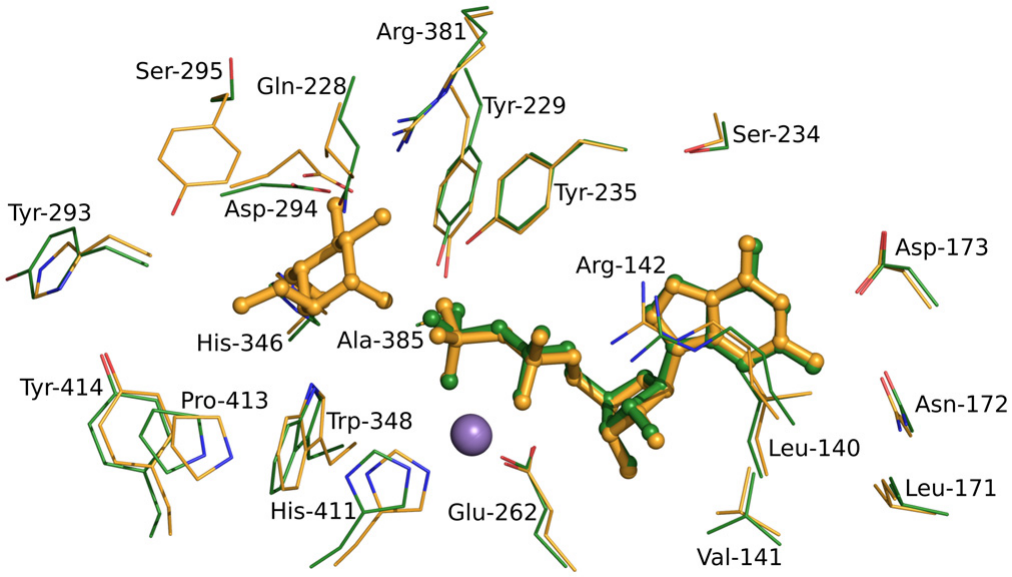
Similarity in the active sites of Kre2p/Mnt1p and Ktr4p. All residues within 5Å of either GDP, Mn^2+^ or methyl-α-mannose are included in the figure and are depicted as lines in green (Ktr4p) or yellow (Mnt1p), while the ligands are depicted as sticks. Residue labels correspond to Ktr4p. Superposition was performed using all C-α atoms of the chain.

While discussing the results of our activity assays, it should be noted that, while the data summarised in Fig 4 does clearly prove that Ktr4p possesses mannosyltransferase activity, the enzyme does not appear to be a particularly efficient catalyst under these assay conditions. This can be attributed to a likely combination of several factors; firstly the assay was performed at room temperature due to experimental limitations, and activity can therefore be expected to be higher at the organism’s optimal growth temperature of 30°C. Secondly, and more significantly, the acceptor substrates we tested are model substrates chosen on the basis that they are readily available commercially. The natural acceptor substrates of the protein would be expected to be much more complex and potentially protein-bound, and it is therefore not at all surprising that the activity of Ktr4p towards these simple non-natural substrates is low. Indeed, the homologue Kre2p/Mnt1p has also been previously shown to display relatively poor activity towards these small substrates [6]. It is also clear from our structure that the large and partially solvent-exposed active site pocket would be capable of accommodating much larger acceptor substrates than those we have tested. Additionally our structure reveals that, although the overall sequence identity of 32% between Ktr4p and Kre2p/Mnt1p is moderate, the active sites of the two enzymes are very highly conserved (Fig 5), which supports our experimental data in suggesting them to have similar functions.

Finally, it is also interesting to compare the relative activities towards the three substrates that were tested. The highest observed activity was towards methyl-α-mannose and activities towards D-mannose and α-1,2-mannobiose, while higher than the control, were significantly lower. Without knowing the natural substrate we can only speculate about the reasons for this. For mannose, which is simplest, least restrained and likely has less possible binding interactions, it might be possible that only a sub-population of the available molecules bind in the correct conformation/orientation for catalysis resulting in a lower affinity and/or catalysis rate. The methyl group of methyl-α-mannose mimics the glycosidic linkage of the extending polysaccharide, introducing restraint and potentially supplying additional interactions in the active site, which could explain the higher activity towards this substrate. In this context it was initially surprising that activity towards α-1,2-mannobiose was not higher. However, the methyl group of methyl-α-mannose is the least bulky substituent possible, while α-1,2-mannobiose includes a bulky second sugar with an α-1,2 linkage when we do not in fact know the identity or linkage of the penultimate sugar in the biological substrate, so this could be suboptimal for catalysis.

Elucidation of the precise function of Ktr4p will require more comprehensive activity assays using biologically active acceptor substrates, in addition to in-cell studies. However, the results presented here represent a major step forward. We show that Ktr4p does indeed possess mannosyltransferase activity, and provide a detailed view of its structure, only the second structure of an enzyme from this family to become available. This work will now provide the basis for future experiments to determine the precise function of the enzyme in the cell.

## Supporting Information

S1 Fig Model of Donor- and Acceptor Sugar Binding. The figure shows the active site and substrate-binding cavity of Ktr4p, with the donor substrate GDP-mannose (in stick representation, with yellow carbons) and an acceptor substrate, mannose (with purple carbons), modelled. Modelling was performed manually in Coot, and followed by cautious use of real-space refinement in the limited density available, which was implemented in Coot. The modelled position of the GDP-mannose was based on our GDP complex structure and information gained from other GT complex structures, as well the observed water molecules of the GDP complex and potential hydrogen-bonding residues in the active site. The acceptor substrate was modelled based on the position of the incomplete density observed in our mannose soaks (the Fo-Fc map, contoured at 3σ, is depicted as green mesh), together with information gained from complex structures of other GT enzymes. The substrate-binding cavity, which extends to the left of the image and is open to the solvent, provides space for much larger acceptor substrates to potentially bind.
(PDF)
