# Supplementary material for: Structure of the Glycosyltransferase Ktr4p from Saccharomyces cerevisiae
Source: PLoS One. 2015 Aug 21;10(8):e0136239. doi: 10.1371/journal.pone.0136239 (PMC4546622; doi:10.1371/journal.pone.0136239)
Supplement: S1 Fig — The figure shows the active site and substrate-binding cavity of Ktr4p, with the donor substrate GDP-mannose (in stick representation, with yellow carbons) and an acceptor substrate, mannose (with purple carbons), modelled. Modelling was performed manually in Coot, and followed by cautious use of real-space refinement in the limited density available, which was implemented in Coot. The modelled position of the GDP-mannose was based on our GDP complex structure and information gained from other GT complex structures, as well the observed water molecules of the GDP complex and potential hydrogen-bonding residues in the active site. The acceptor substrate was modelled based on the position of the incomplete density observed in our mannose soaks (the Fo-Fc map, contoured at 3σ, is depicted as green mesh), together with information gained from complex structures of other GT enzymes. The substrate-binding cavity, which extends to the left of the image and is open to the solvent, provides space for much larger acceptor substrates to potentially bind. (PDF) [file pone.0136239.s001.pdf]

Supplementary Figure 1

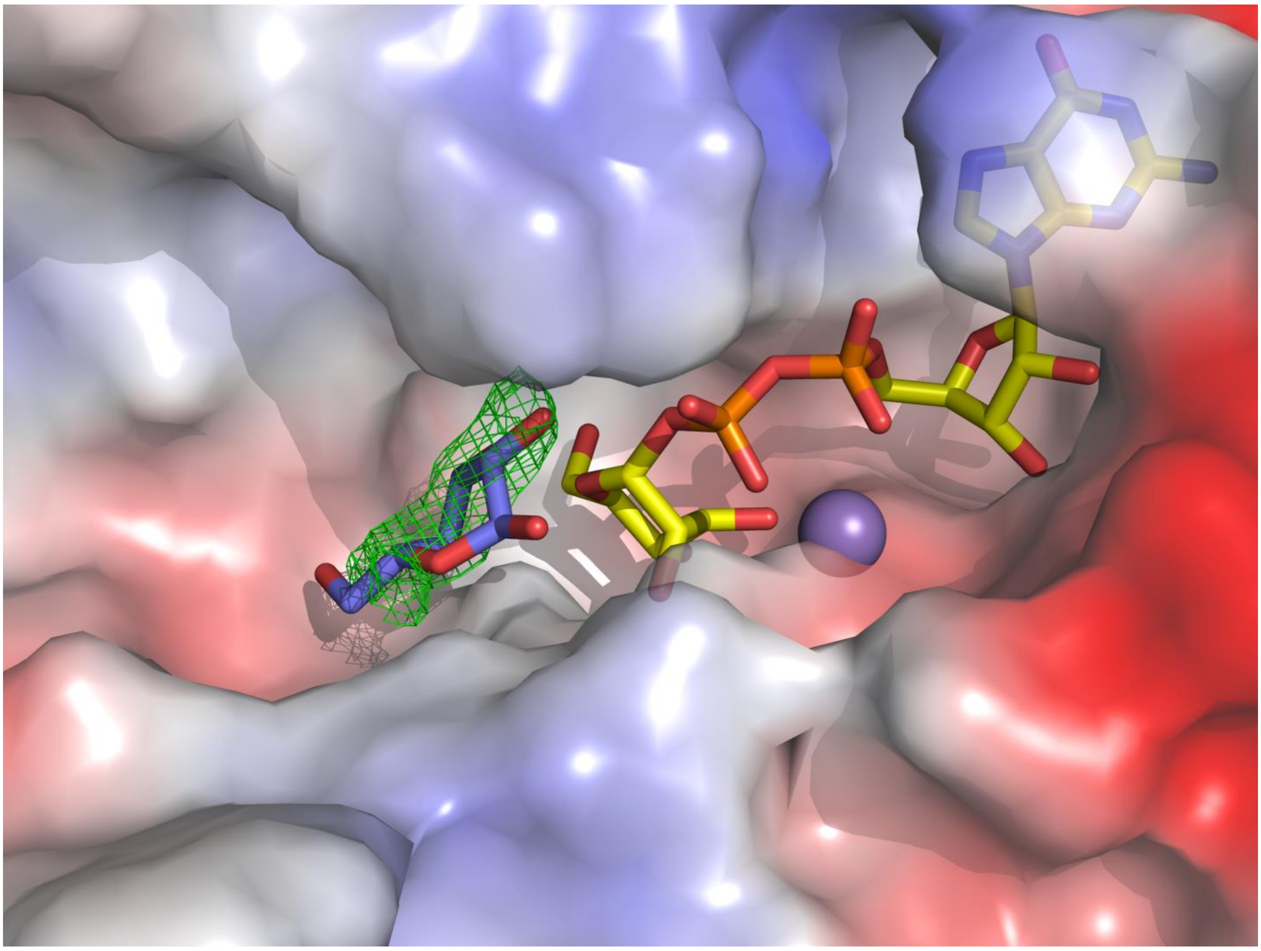

Figure legend: The figure shows the active site and substrate-binding cavity of Ktr4p, with the donor substrate GDP-mannose (in stick representation, with yellow carbons) and an acceptor substrate, mannose (with purple carbons), modelled. Modelling was performed manually in Coot, and followed by cautious use of real-space refinement in the limited density available, which was implemented in Coot. The modelled position of the GDP-mannose was based on our GDP complex structure and information gained from other GT complex structures, as well the observed water molecules of the GDP complex and potential hydrogen-bonding residues in the active site. The acceptor substrate was modelled based on the position of the incomplete density observed in our mannose soaks (the Fo-Fc map, contoured at 3σ, is depicted as green mesh), together with information gained from complex structures of other GT enzymes. The substrate-binding cavity, which extends to the left of the image and is open to the solvent, provides space for much larger acceptor substrates to potentially bind.
